# Data and model tango to aid the understanding of astrocyte-neuron signaling

**DOI:** 10.3389/fncom.2014.00003

**Published:** 2014-01-24

**Authors:** Shivendra Tewari, Vladimir Parpura

**Affiliations:** ^1^Department of Molecular and Integrative Physiology, University of MichiganAnn Arbor, MI, USA; ^2^Department of Neurobiology, University of Alabama at BirminghamBirmingham, AL, USA; ^3^Department of Biotechnology, University or RijekaRijeka, Croatia

**Keywords:** astrocytes, neurons, signaling, tripartite synapse, mathematical model

**Figure 1:**
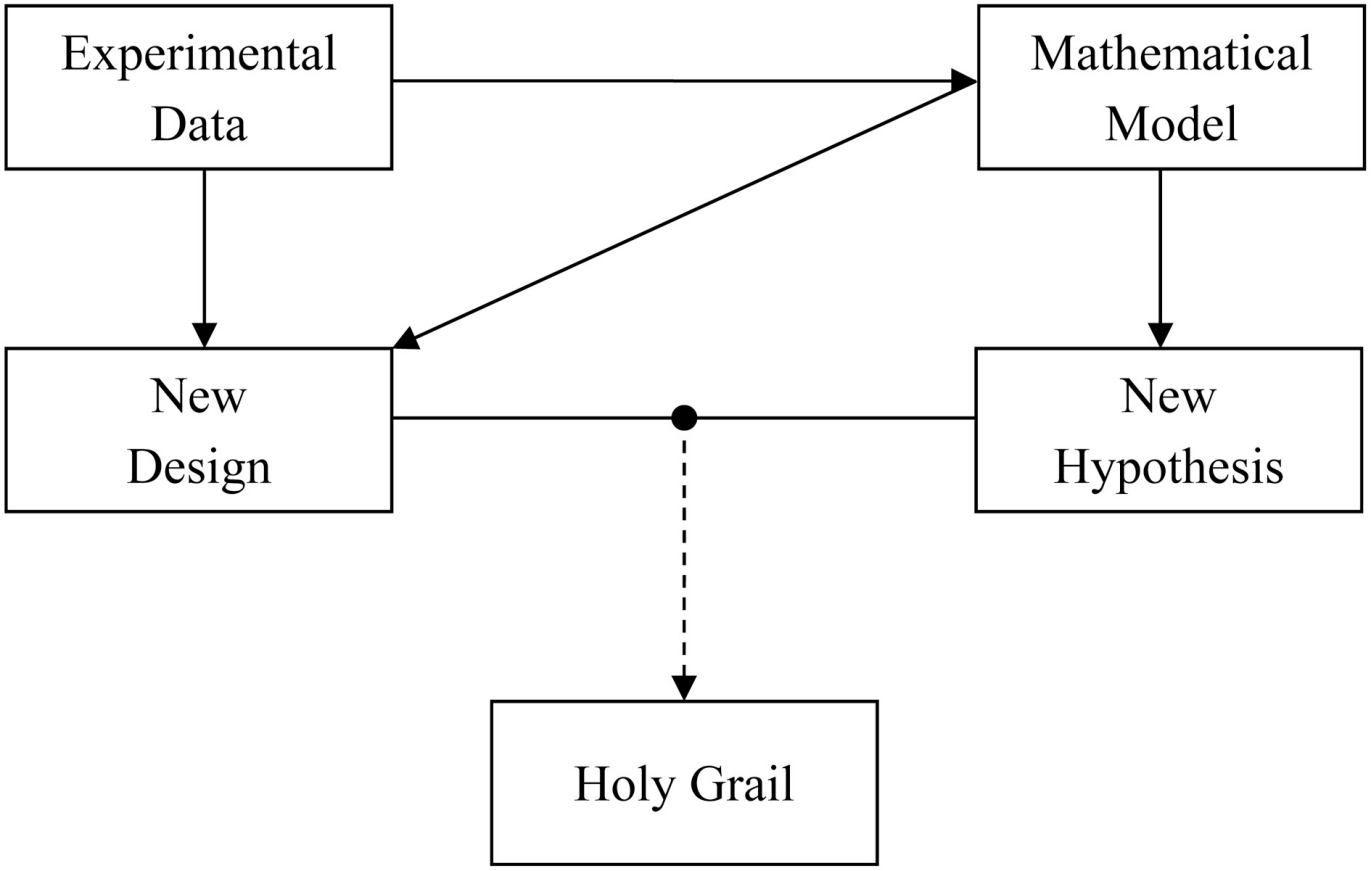
**The “tango” between experimental data and mathematical model is necessary for a faster and deeper understanding of the role of astrocyte-neuron signaling in the brain**. Experimental data are important for signaling pathway quantification using mathematical models. The experimental data in turn can gain from the model by testing a hypothesis *in silico* or designing new experiments to test the hypothesis *in vivo* or *in vitro*. It is a combination of both the approaches that could lead us to a wholesome understanding of the human brain, i.e., the holy grail of neuroscience.

Alan Lloyd Hodgkin and Andrew Fielding Huxley revolutionized the field of neuroscience when they provided a quantitative description of the squid axon action potential generation and propagation (Hodgkin and Huxley, 1952). The seminal article described how to recreate a biological phenomenon inside a computer using a mathematical model. Ever since, this quantitative approach has helped us in providing a deeper and better understanding of the experimental system. That being said, it is the experimental data which generates new science, while mathematical models basically work as a tool that provides description of the same data across different scales and planes. We briefly discuss a data-model pair “tango” (Figure 1) through an example of astrocyte-neuron signaling.

The work done in 1990s demonstrated that astrocytes, a type of glial cells, can actually talk to the surrounding neurons by releasing glutamate in a similar manner as neurons do (Parpura et al., 1994). This glutamate-mediated astrocyte-neuron signaling was found to modulate synaptic transmission leading to the concept of the tripartite synapse (Araque et al., 1999), a *triumvirate* in which the peri-synaptic astrocytes enwrap pre- and post-synaptic neural elements, and play a dynamic role in listening and talking to the neuronal signaling transfer. However, the experiments investigating astrocyte-neuron signaling are not trivial. They are technically challenging as astrocytes, unlike neurons, are electrically non-excitable. Moreover, the visualization of a tripartite synapse in physiological conditions is hampered by the imposing limits of light microscopy. Despite these limitations, a significant amount of revelations have been made. Hence, astrocytes can listen to the pre-synaptic neuron firing, i.e., glutamate release (Porter and McCarthy, 1996), and can signal the neurons by releasing chemical transmitters (e.g., glutamate, D-serine, and ATP), which includes the induction of synaptic plasticity, i.e., short-term and long-term potentiation (Pascual et al., 2005; Perea and Araque, 2007; Henneberger et al., 2010; Navarrete et al., 2012), and short-term depression (Andersson and Hanse, 2010), as well as the synchronization of the electrical activity of surrounding neurons (Fellin et al., 2004). While enormously informative, these individual pieces of information observed during different experiments can hardly offer a gestalt viewpoint of the operation of the brain and the role of the tripartite synapse and astrocyte-neuron signaling in it. What happens when we combine these pieces of the puzzle and what might the solution look like? Which pieces contribute more than the others? These are a few questions to which obtaining answers using an experimental approach can be extremely difficult and time consuming, as it would involve the integration of synaptic details at a scale of few hundreds to many thousands of tripartite synapses. Namely, a single astrocyte can cover 20–100 thousand synapses in rodents and up to 2 million synapses in primates and humans (Bushong et al., 2002; Oberheim et al., 2006; Verkhratsky et al., 2011).

But, with the aid of computational modeling, it is, indeed, possible to answer some of the questions that we just posed. In fact, some of the questions related to the tripartite synapse have already been addressed using computational models. Nadkarni and Jung (Nadkarni and Jung, 2003) were the first to study the effect of astrocyte-induced currents on synaptic transmission. Their quantitative model was based on the experimental data (Parpura and Haydon, 2000), which entails the astrocytic Ca^2+^ levels necessary and sufficient to cause glutamate release from these glial cells, and can induce slow inward currents in the surrounding neurons. Using a relatively simple mathematical model, they revealed a possible role of astrocytes in temporal lobe epilepsy, for which only circumstantial evidence was available. Interestingly, a later experimental study (Tian et al., 2005) discovered that astrocytic currents can, indeed, induce epileptiform activity in cortical neurons.

With the advent of technology, many fine details about astrocyte-neuron signaling have been discovered which were not available or accounted for by earlier models of the tripartite synapse (Volman et al., 2007; Nadkarni et al., 2008). Recently, Tewari and Majumdar (2012) developed a comprehensive and biologically realistic model of a tripartite synapse, which accounted for the vesicular glutamate release from an astrocyte via Ca^2+^-dependent exocytosis (Montana et al., 2006; Malarkey and Parpura, 2011). Using this detailed model, they could study the effect of astrocytic metabotropic glutamate receptor 5 (mGluR5) expression on astrocyte-induced short-term plasticity at single hippocampal synapse. Their model predicted that the astrocytic mGluR5 expression is a potent regulator of astrocyte-induced synaptic plasticity, and provided a quantitative framework under which astrocytes do not potentiate synapses. In conjunction, it has been experimentally demonstrated that the expression of mGluR5 is age-dependent (Cai et al., 2000; Sun et al., 2013). Combined model-data findings of the above studies would predict that under certain experimental conditions astrocytes do not potentiate synapses. Serendipitously, such predicted experimental conditions were matched in a study observing a lack of evidence for the role of astrocytes in short- and long-term synaptic potentiation (Agulhon et al., 2010).

More recently, Tewari and Parpura (2013) studied the effects that an astrocyte has on a pack of four CA1 pyramidal neurons synapsed by a CA3 neuron Schaffer collateral. This computational model confirmed the experimental findings, described elsewhere (Fellin et al., 2004), that astrocytes can synchronize neuronal electrical activity. Additionally, astrocytes affected neuronal firing pattern, which attained a delta rhythm, indicating a potential role of astrocytes in contextual learning and memory. However, whether such a phenomenon exists in a living organism remains to be experimentally verified.

Taken together, the above mentioned models have contributed to an elucidation and advancement of the concept of astrocyte-neuron signaling. With an advent of enhanced computing resources and quantitative modeling, computational contributions to the acquisition of new knowledge, cross-checking on experimental data, and proposing new venues for experimentalists (Figure 1) are only expected to grow. Naturally, modeling approach is highly efficient in reducing the total usage of animals. As both the approaches, experimental, and computational, have their limitations, it is, hopefully, a combined data-model approach that could result in a wholesome understanding of astrocyte-neuron interactions and by extension of the human brain, i.e., finding the holy grail of neuroscience.
